# Evolutionary insight on localization of 18S, 28S rDNA genes on homologous chromosomes in Primates genomes

**DOI:** 10.3897/CompCytogen.v12i1.19381

**Published:** 2018-01-24

**Authors:** Sofia Mazzoleni, Michail Rovatsos, Odessa Schillaci, Francesca Dumas

**Affiliations:** 1 Dipartimento di Scienze e Tecnologie Biologiche, Chimiche e Farmaceutiche (STEBICEF), Università degli Studi di Palermo, via Archirafi 18; 2 Faculty of Science, Department of Ecology, Viničná 7, Charles University, Pragha 2, Czech Republic

**Keywords:** Fluorescence *in situ* hybridization, repetitive DNAs, synapomorphy, Primates, tree shrew

## Abstract

We explored the topology of 18S and 28S rDNA units by fluorescence *in situ* hybridization (FISH) in the karyotypes of thirteen species representatives from major groups of Primates and *Tupaia minor* (Günther, 1876) (Scandentia), in order to expand our knowledge of Primate genome reshuffling and to identify the possible dispersion mechanisms of rDNA sequences. We documented that rDNA probe signals were identified on one to six pairs of chromosomes, both acrocentric and metacentric ones. In addition, we examined the potential homology of chromosomes bearing rDNA genes across different species and in a wide phylogenetic perspective, based on the DAPI-inverted pattern and their synteny to human. Our analysis revealed an extensive variability in the topology of the rDNA signals across studied species. In some cases, closely related species show signals on homologous chromosomes, thus representing synapomorphies, while in other cases, signal was detected on distinct chromosomes, leading to species specific patterns. These results led us to support the hypothesis that different mechanisms are responsible for the distribution of the ribosomal DNA cluster in Primates.

## Introduction

Repetitive DNA elements make up a large portion of eukaryotic genomes and include tandem arrays and dispersed repeats. These genomic components are able to change the molecular composition of chromosomes and their study will contribute to the knowledge of karyotype differentiation (Cioffi et al. 2010, Dumas et al. 2017). A prominent repetitive DNA element organized in tandem repetition consists of ribosomal DNA (rDNA) encoding the ribosomal RNA, essential for cell function. The rDNA region is divided into two families: the 5.8S (minor) and the second one the 45S (major) comprising 18S and 28S loci. The chromosome regions with transcriptionally active 45S loci, referred as the Nucleolus Organizer Regions (NORs), can be identified either by silver staining (Ag-NOR) or, more accurately, by fluorescence in situ hybridization (FISH) which permits researchers to identify both inactive and active NORs. rDNA probes have been cytogenetically mapped by FISH in the karyotypes of several vertebrate species, representatives of fishes (Srikulnath et al. 2009, 2011, Sember et al. 2015), reptiles (Rovatsos et al. 2015a, 2015b, 2016), and Artiodactyla (Nguyen et al. 2008, Degrandi et al. 2014), rodents (Gornung et al. 2011, Cazaux et al. 2011, Britton-Davidian et al. 2012) and bats (Calixto et al. 2014) in mammals in order to clarify their chromosomal location and mechanisms of dispersion. The topology of rDNA loci is widely used as marker for comparative cytogenetic studies and to explore evolutionary relationships, since such loci often show species-specific patterns (Srikulnath et al. 2009, 2010, Cazaux et al. 2011, Bulatova and Pavlova 2016). Furthermore, the variation in number and topology of rDNA genes has been shown at inter- and intra-species levels, explained as consequence of chromosomal rearrangements, ectopic recombination through association of rDNA with other chromosomal segments during meiotic division or transposition events (Hirai et al. 1996, Eickbush and Eickbush 2007, Baicharoen et al. 2016).

Concerted evolution of rDNA clusters caused by unequal cross over is a well-documented process; rDNA gene copies within an individual and within a species remain identical in sequence, while between closely related species the sequence can vary widely (Averbeck and Eickbush 2005). In humans, it has been demonstrated that the dynamic length variation occurring at rDNA clusters, is the direct result of unequal cross over occurring both inter- and intrachromosomally (Stults et al. 2008). Recently it has been showed that highly degraded, but near full length, rDNA units can be found at multiple sites in the human genome chromosomes. These sequences tend to accumulate close to centromeres and to change from canonical rDNA to pseudogenes, representing different stages in the evolution of the rDNA sequences (Robicheau et al. 2017).

rDNA distribution especially of the 18S and 28S loci has been investigated in many species of Primates either by FISH (Henderson et al. 1974a,b, 1976, 1977, 1979, Hirai et al. 1999, 2007, Guillén et al. 2004, Baicharoen et al. 2016) or silver staining (Tantravahi et al. 1976, Bedard et al. 1978, Masters et al. 1987, Nagamachi et al. 1992, Hirai et al. 2007, Tanomtong et al. 2009), including *Homo sapiens* Linnaeus, 1758. In humans, NORs have been identified on the secondary constriction of five pairs of acrocentric chromosomes: 13, 14, 15, 21 and 22 (Henderson et al. 1972, Tantravahi et al. 1976).

In pioneering comparative studies on Primates, it was assumed that there is no homology between chromosomes bearing rDNA (Henderson et al. 1977). Furthermore, exchanges among rDNA genes on non-homologous chromosomes (Arnheim et al. 1980) and a multiple topologies of rDNA sites with species-specific variations (Hirai et al. 1999) have been shown in Hominoidea. Later, intra-species polymorphisms have also been described in Primates such as *Pan troglodytes* (Blumenbach, 1775) (Guillén et al. 2004), *Hylobates lar* (Linnaeus, 1771), (Tanomtong et al. 2009) and *Nycticebus bengalensis* (Geoffroy, 1812) (Baicharoen et al. 2016), possibly related to unequal crossing over or to transcriptional inactivation by methylation of NORs.

Therefore, we tried to explore the chromosomal distribution of rDNA loci in Primate genomes, by mapping the 18S and 28S probe in thirteen species of Primates and in *Tupaia minor* (Günther, 1876), the representative of the order Scandentia, as outgroup (Lin et al. 2014, Zhou et al. 2015). The chromosome topology of rDNA genes by FISH has been analyzed in a wide phylogenetic framework taking in consideration previous literature.

## Material and methods

The Primates species analyzed through rDNA probes mapping are listed in Table 1. In the present work, rDNA distribution is documented by FISH analysis for the first time in ten species and hybridization was repeated for *Hylobates lar, Lemur catta* (Linnaeus, 1758) and *Symphalangus syndactylus* (Raffles, 1821) formerly studied (Warburton et al. 1975, Henderson et al. 1977, Hirai et al. 1999). Metaphases for all species have been obtained following the standard protocol (Sineo et al. 2007, Small et al. 1985), from primary cultures of fibroblast cell lines treated and fixed at the National Cancer Institute, USA by F. Dumas and R. Stanyon. All karyotypes have been analyzed after DAPI inverted banding. The probe for the rDNA sequence was prepared from a plasmid (pDmr.a 51#1) with a 11.5-kb insert encoding the 18S and 28S ribosomal units of *Drosophila melanogaster* (Meigen, 1830) (Endow 1982), and it was subsequently labelled with biotin-dUTP using a Nick Translation Kit (Abbott). In situ hybridization of the probe with the chromosomal spreads was performed overnight according to standard protocol and the probe signal was enhanced and detected using an avidin-FITC/biotinylated anti-avidin system (Vector Laboratories) (Rovatsos et al. 2015a). Probe signals have been pseudocolored in red for better contrast. The chromosomes were counterstained with DAPI, and the slides were mounted with antifade medium Fluroshield (Sigma-Aldrich).

**Table 1.** List of species (Primates, Scandentia) studied cytogenetically with rDNA probes mapped by FISH; the chromosomes pairs bearing rDNA probe signals and the human homologies (HSA) identified through the analysis of the painting references are reported. A - acrocentric, SM - submetacentric, C - centromere. * - FISH markers position in human synteny association. HSA homology was extrapolated for *Otolemur garnettii* (OGR#) from *O. crassicaudatus* Géoffroy, 1812 G-banding data (Masters et al. 1987) since they showed close karyotypes.

Species	rDNA mapping				HSA homologs		Painting References
	Chr.	Chromosome type	Position	2ndary constriction			
**Strepsirrhini**							
*Lemur catta* *LCA* (Linnaeus, 1758)	21	Acrocentric	Centromere	No		22/12	Cardone et al. 2002
	25	Acrocentric	Centromere	No		8	
*Otolemur garnettii* *OGR* (Ogilby, 1838)	19	Acrocentric	Centromere	No		17	Stanyon et al. 2002*
Platyrrhini							
*Callithrix jacchus* *CJA* (Linnaeus, 1758)	15	Acrocentric	Centromere	No		3	Neusser et al. 2001
	17	Acrocentric	Centromere			3	
	19	Acrocentric	Centromere			1	
*Callimico goeldii* *CGO* (Thomas, 1904)	14	Acrocentric	Centromere	No		5	Neusser et al. 2001
	15	Acrocentric	Centromere	No		*9/22	
	16	Acrocentric	Centromere	No		*15/3	
	17	Acrocentric	Centromere	No		*13/17	
	21	Acrocentric	Centromere	No		20	
	22	Acrocentric (only in 1 homologous)	Centromere	No		*3/21	
*Saguinus Oedipus* *SOE* (Linnaeus, 1758)	20	Acrocentric	q arm	No		1	Neusser et al. 2001
	21	Acrocentric	q arm	No		1	
	22	Acrocentric	q arm	Yes		10	
*Saimiri sciureus* *SSC* (Linnaeus, 1758)	6	Submetacentric	Centromere	Yes		20/3	Stanyon et al. 2000
*Ateles paniscus paniscus* *APA* (Linnaeus, 1758)	8	Submetacentric	Centromere/q arm	Yes		19/*20	de Oliveira et al. 2005
*Alouatta caraya* *ACA* (Humboldt, 1812)	17	Acrocentric	q arm	Yes		8	de Oliveira et al. 2002
	23	Acrocentric	q arm	Yes		1	
**Catarrhini**							
*Chlorocebus aethiops* *CAE* (Linnaeus, 1758)	19	Subtelomeric	Centromere/q arm	Yes		22	Finelli et al. 1999
*Colobus guereza* *CGU* (Rüppell, 1835)	16	Submetacentric	Centromere/q arm	Yes		22/21	Bigoni et al. 1997
*Erythrocebus patas* *EPA* (Schreber, 1774)	26	Submetacentric	Centromere	No		22	Stanyon et al. 2005
*Hylobates lar* *HLA* (Linnaeus, 1771)	12	Submetacentric	q arm	Yes		2*/*3	Jauch et al. 1992
*Symphalangus syndactylus* *SSY* (Raffles, 1821)	21	Acrocentric	Centromere	No		3	Muller et al. 2003
	Y	Acrocentric	Centromere	No		Y	
**Scandentia**							
*Tupaia minor* *TMI* (Günther, 1876)	25	Acrocentric	Centromere	No		3	Dumas et al. 2012
	26	Acrocentric	Centromere	No		9	
	28	Acrocentric	Centromere	Yes		12*/*22	

Karyotypes were examined by inverted DAPI method, as previously performed (Dumas et al. 2016, Mazzoleni et al. 2017); the human homology between chromosomes with rDNA signal was identified based on painting data from previous projects (Table 1). Our data have been compared with previous literature data on rDNA mapping in Primates (Table 2). The results of distribution of rDNA loci on the chromosomes of all analyzed species are illustrated in a graphical reconstruction of the primate phylogenetic tree, following Perelman and colleagues (2011) with some modification, created by MESQUITE v.2.75 (Maddison and Maddison 2011).

**Table 2.** List of Primates - Scandentia species analyzed with the mapping data from rDNA probes and the respective references.

Species	rDNA mapping references
Catarrhini	
*Colobus polykomos*	Henderson et al. 1977
*Gorilla gorilla*	Henderson et al. 1976; Hirai et al. 1999
*Hylobates agilis*	Hirai et al. 1999
*Hylobates lar*	Warburton et al. 1975
*Hylobates* × *Nomascus hybrid*	Hirai et al. 2007
*Macaca fuscata fuscata*	Hirai et al. 1998
*Macaca mulatta*	Henderson 1974a
*Pan paniscus*	Henderson et al. 1976; Hirai et al. 1999
*Pan troglodytes*	Henderson 1974b; Hirai et al. 1999; Guillén et al. 2004
*Pongo pygmaeus albei*	Henderson et al. 1979
*Papio cynocephalus*	Henderson et al. 1977
*Papio hamadryas*	Henderson et al. 1977
*Symphalangus syndactylus*	Henderson et al. 1976; Hirai et al. 1999
Platyrrhini	
*Ateles geoffroyi*	Henderson et al. 1977
*Pithecia pithecia*	Henderson et al. 1977
*Saguinus nigricollis*	Henderson et al. 1977
Strepsirrhini	
*Lemur fulvis*	Henderson et al. 1977
*Nycticebus bengalensis*	Baicharoen et al. 2016

## Results

FISH signals were located in different positions on primarily small particular chromosomes of taxa studied. The variation was observed between karyotypes regarding both the number and morphology of chromosomes bearing the signal as the rDNA site number per karyotype.

**Figure 1.**
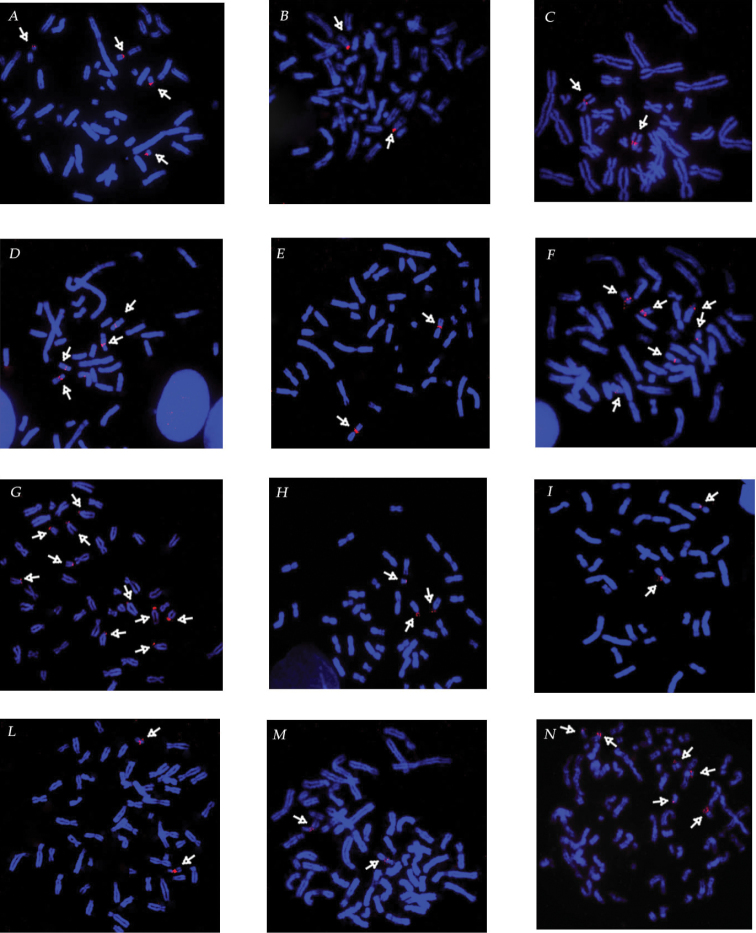
rDNA loci mapping (red signal highlighted by white arrows) on metaphases of: **A** *Lemur catta* **B** *Otolemur garnettii* **C** *Ateles paniscus paniscus* **D** *Alouatta caraya* **E** *Saimiri sciureus* **F** *Saguinus oedipus* **G** *Callimico goeldii* **H** *Symphalangus syndactilus* **I** *Hylobates lar* **L** *Chlorocebus aethiops* **M** *Erythrocebus patas* **N** *Tupaia minor*.

**Figure 2.**
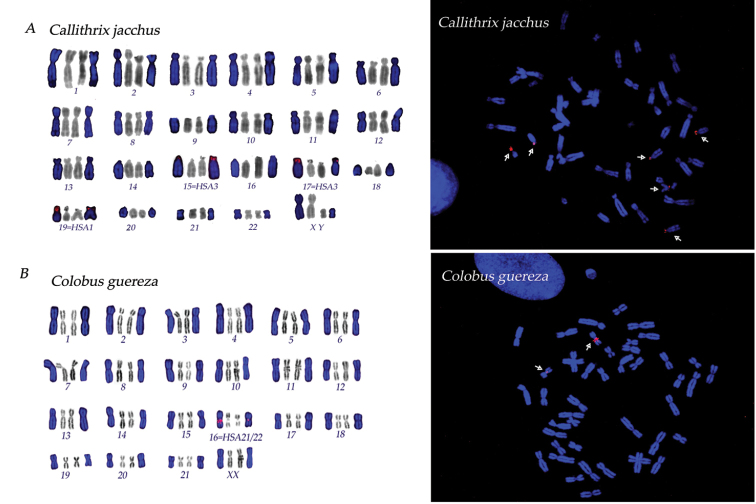
DAPI stained chromosomes (blue) with rDNA loci signal (red) are illustrated, together with DAPI inverted (grey) chromosomes arranged in karyotypes of **A** *Callithrix jacchus* **B** *Colobus guereza*. Corresponding metaphases (with red signals highlighted by white arrows) are shown on the left.

From one to five rDNA autosome markers were located at the tip of acrocentrics in 5 species: *Lemur catta* (pairs 21, 25) (Fig. 1A), *Otolemur garnettii* Ogilby, 1838, (pair 19) (Fig. 1B), *Callithrix jacchus* Linnaeus, 1758, (pairs 15, 17, 19) (Fig. 2A), *Callimico goeldii* Thomas, 1904, (pairs 14-17, 21 and, not frequent, 22 – single homolog) (Fig. 1G) and *Symphalangus syndactylus* (pair 21 and the Y-chromosome) (Fig. 1H).

In 7 species, pericentromeric position was recorded for a biarmed pair: *Saimiri sciureus* Linnaeus, 1758 (submetacentrics pair 6) (Fig. 1E), *Ateles paniscus paniscus* Linnaeus, 1758, (pair 8) (Fig. 1C), *Hylobates lar* (pair 12) (Fig. 1I), *Colobus guereza* Rüppell, 1835, (pair 16) (Fig. 2B), *Saguinus Oedipus* Linnaeus, 1758, (pair 20) (Fig. 1F), *Erythrocebus patas* Schreber, 1775, (pair 26) (Fig. 1M), or subtelocentric chromosomes 19 of *Chlorocebus aethiops* Linnaeus, 1758,(subtelocentric chromosomes 19) (Fig. 1L). Besides, in *Saguinus oedipus* the location on acrocentrics 21 and 22 was identified in a visible secondary constriction (Fig. 1F).

In *Alouatta caraya* Humboldt, 1812, signals were positioned on medium-small acrocentrics with a visible secondary constriction (pairs 17, 23) (Fig. 1D). Similarly, three small acrocentrics of *Tupaia minor* were marked (pairs 25, 26, 28) (Fig. 1N).

**Figure 3.**
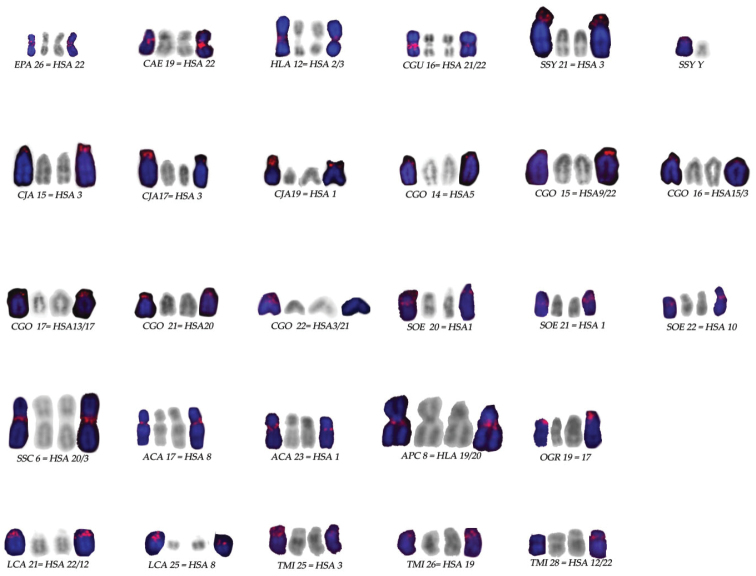
Chromosome pairs bearing rDNA probe signals for each species analyzed and corresponding human syntenies (HSA): chromosomes are in DAPI inverted banding; rDNA probe signals in red.

The results are reported also in Figure 3 and summarized in Table 1. Homology between marked chromosomes is below discussed.

## Discussion

rDNA mapping has been previously performed in a number of Primate species (Table 2), but in pioneering studies, the cross-species homology of chromosomes with rDNA could not be reliably identified due to limitations of G-banding and the lack of advanced molecular cytogenetic methods, such as chromosome painting. For example, the topology of rDNA loci was previously studied in *Hylobates lar*, *Lemur catta* and *Symphalangus syndactylus* (Warburton et al. 1975, Henderson et al. 1977, Hirai et al. 1999), but at that time, it was not always possible to identify the hybridized chromosomes nor their homology with human chromosomes. In our study, we were able to identify, in all studied species, the homology and synteny of each chromosome bearing rDNA loci to human karyotype, through DAPI inverted banding.

**Figure 4.**
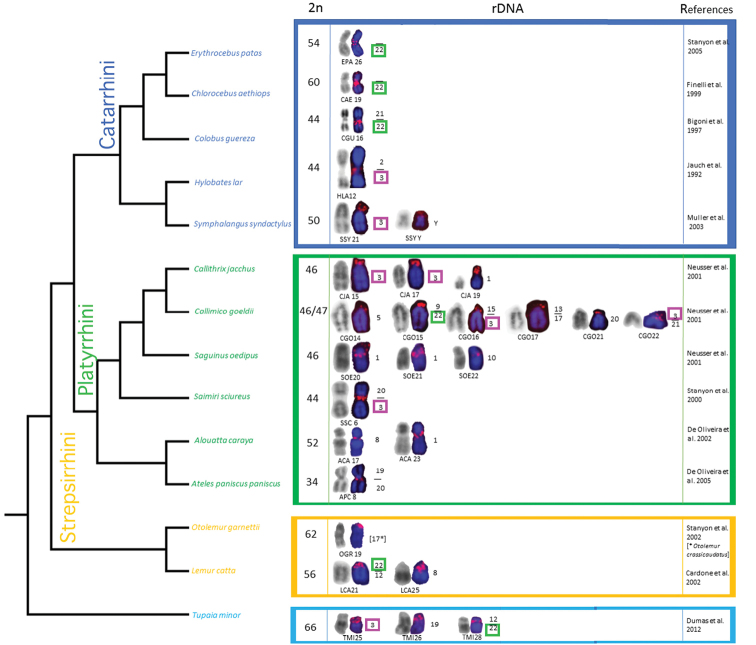
Primate molecular phylogenetic relationships as modified after Perelman et al. (2011). The tree was reconstructed in MESQUITE in consideration of the diploid number (2n), the DAPI stained chromosome (blue) with the rDNA probe signals localization (red) and the inverted DAPI (grey) for each species. In each chromosome pair, only a single chromosome is shown. Homologies to human chromosomes are indicated on the right side of chromosomes and are inferred through the analysis of the references listed in the last column. Ancestral localization of rDNA loci is underlined in color: green for human synteny 22, pink for human synteny 3.

The data concerning the distribution of rDNA loci on the chromosomes of the analyzed species are discussed in an evolutionary perspective and illustrated in a graphical reconstruction (Fig. 4) based on chromosome characters such as is visualized in the tree; we report for each species the diploid number, rDNA-bearing chromosomes and the homology to human syntenies.

The comparative analysis of ours and other data demonstrated that rDNA loci are often localized in the chromosomes homologous to HSA synteny 3 and 22 in many Primates and in *Tupaia* as well (Fig. 3). Indeed, among Primates, we found the rDNA loci on HSA synteny 3 on Platyrrhini species *S. sciureus*, *C. jacchus*, *C. goeldii* and in gibbons *H. lar* and *S. syndactilus*. In addition, data from literature on the Prosimian *Nycticebus bengalensis* Lacepede, 1800, (Baicharoen et al. 2016) show that rDNA loci exist on human synteny 3. Furthermore, we identified rDNA loci on HSA synteny 22 in the Prosimian representative *L. catta*. Similar topology of rDNA loci was presented previously in *N. bengalensis* (Baicharoen et al. 2016). Among Platyrrhini, even if the probe localized at the centromere of *C. goeldii* chromosome 15, close to human synteny 9, this last synteny is associated to human synteny 22, thus leading us to propose the hypothesis that an inversion could have relocated it after the fusion of the two involved syntenies. In all Cercopithecoidea studied (*C. aethiops, C. guereza* and *E. patas*), the rDNA loci were localized on human synteny 22; in *C. guereza* where it is between syntenies 22 and 21 presumably it conserved its position after the fusion of the first chromosome bearing the rDNA with the second one; other data from literature indicate that rDNA localized on human synteny 22 also in Hominoidea species such as *Pan paniscus* (Schwarz, 1929), *P. troglodytes*, *Gorilla gorilla* (Geoffroy, 1852) and in *H. sapiens* (Linnaeus, 1758) (Hirai et al. 1999, Tantravahi et al. 1976, Guillén et al. 2004). These results led us to suppose that rDNA on synteny 3 and 22 represents the ancestral status; presumably rDNA on synteny 3 has been lost in prosimians (LLC, OGR), Cercopithecoidea (CAE, CGU, EPA) and in many Platyrrhini, while the rDNA on synteny 22 has been lost in gibbons (HLA, SSY).

Other multiple rDNA signals that we detected on different chromosomes, could be apomorphies with species specific locations such as, for example, the one found on chromosomes homologous to human synteny 17 in *O. garnettii*. Consistent with previous findings in *N. bengalensis* our data well correspond to species specific rDNA locations (Baicharoen et al. 2016). Furthermore, other rDNA loci could represent synapomorphisms in closely related species, such as the ones on HSA synteny 1 in *S. oedipus*, *A. caraya* and *C. jacchus* (Platyrrhini), as well as on HSA synteny 13/14 previously shown in Hominoidea (*Pan troglodytes*, *P. paniscus*, *H. sapiens*) (Tantravahi et al. 1976, Henderson et al. 1976, Hirai et al. 1999).

Despite the facts that have documented a conserved pattern in the topology of rDNA loci in many species (e.g. extensive homology to HSA synteny 3 and 22), we also showed the presence of multiple rDNA loci on distinct chromosomes (Fig. 4). Therefore, we assume that different mechanisms are responsible for their dispersion in genome, in agreement with previous hypotheses (Hirai et al. 1999, Britton-Davidian et al. 2012). We conclude that intra- and interchromosomal rearrangements are probably not the single explanation of the rDNA pattern in Primates. Ectopic recombination might be responsible for the gain and loss of rDNA loci, resulting in the dispersal or loss of rDNA tandem repeats during meiosis, more prone to occur at the terminal tip of acrocentric chromosomes. For example, among the studied Primates, we found multiple topologies with up to five pairs of acrocentric chromosomes carrying the rDNA loci in *C. goeldii* (Platyrrhini). Actually, the similarity of five to eight pairs has been previously reported in literature for human (Henderson et al. 1972, Tantravahi et al. 1976), chimpanze and gorilla (Hirai et al. 1999).

In an alternative view, we cannot exclude the case that short tandem repeats of rDNA loci may exist on multiple chromosomes, beyond the detection efficiency of FISH, which were inherited by the ancestors of the extant Primates, and were subsequently amplified independently in different species during the evolution of their karyotypes, resulting in the extensive variability observed in this study. Concluding, our results indicate that rDNA distribution is due to different mechanisms; we found species with conserved signals on syntenic chromosomes, while in others, signal was detected in distinct chromosomes. There are reasons to pay more attention to the study of rDNA loci in Primates chromosomes as marks of the complex evolutionary relationships.
